# Integrating AI-Driven Diagnostics in Arrhythmia Care to Enhance Patient Outcomes: A Narrative Review

**DOI:** 10.7759/cureus.113138

**Published:** 2026-07-22

**Authors:** Mustafa Abrar Zaman, Kuragamage Dona Prabuddhi Thiloka Kuragama, Aseel Aljeshi, Mikhail Johnson, Jyothsnaa Sathish, Kush J Kanjia, Adhved Krishnan, Ronaldo Sabbagh, Aliea N Ramjag, Rayan Rezaei, Sweety Akter, Fariha Abdullah, Numan Baydemir

**Affiliations:** 1 Cardiology, St. George's University School of Medicine, St. George's, GRD; 2 Surgery, St. George's University School of Medicine, St. George's, GRD; 3 Anesthesiology, St. George's University School of Medicine, St. George's, GRD; 4 Emergency Medicine, St. George's University School of Medicine, St. George's, GRD; 5 Biochemistry, St. George's University School of Medicine, St. George's, GRD; 6 Gynecology, St. George's University School of Medicine, St. George's, GRD; 7 Biotechnology, BRAC (Bangladesh Rural Advancement Committee) University, Dhaka, BGD; 8 Medicine, North Middlesex University Hospital NHS Trust, London, GBR

**Keywords:** ai diagnostics, arrhythmia, artificial intelligence, cardiovascular disease, deep learning, ecg interpretation, machine learning, patient care, predictive modeling, wearable technology

## Abstract

International morbidity and mortality are led by cardiovascular disease, specifically arrhythmias. The integration of artificial intelligence (AI) can promote earlier identification and, therefore, provide personalized clinical judgments sooner, which can avert these consequences. AI is mainly used in diagnostics, prognostics, and decision support through test interpretations (electrocardiographs (ECGs) and MRI scans) and computing hidden characteristics to utilize a personalized plan rather than a population-based one. While such technology is available in hospitals, some functions are also being integrated into smart wearable devices. These wearable devices allow for continuous monitoring rather than limiting it to in-hospital settings, thus making it easier to enable an earlier diagnosis. However, certain arrhythmias, such as asymptomatic arrhythmias, can go unnoticed by AI. Additionally, there are questions regarding data transparency, privacy, and the financial burden of utilizing and managing AI in medical settings.

## Introduction and background

Cardiac arrhythmias contribute significantly to cardiovascular disease, as they are the leading cause of morbidity and mortality in heart failure, sudden cardiac death, and stroke worldwide. Traditionally, reviewing electrocardiograph (ECG) records with the assistance of a healthcare professional is considered the foundation for cardiac arrhythmia diagnosis [1]. However, limitations such as reduced monitoring duration, variability among clinicians, and reduced sensitivity for paroxysmal or asymptomatic arrhythmias delay and hinder the accuracy of diagnosis [2]. To improve cardiovascular care with the aid of artificial Intelligence (AI) and technology, these challenges highlight the need for more precise diagnostic strategies. AI is a vital component of modern medicine that aids in identifying complex components in medicine within a large scope of clinical data and information. In cardiology, AI is used for various modes of screening and diagnosis, including ECG, imaging, genomics, and electronic health records. Thus, AI supports the early identification of diseases, thereby enhancing individualized prognostic determinants. Recent developments in machine learning (ML) and deep learning (DL) have widened the use of AI from automated interpretation to predictive and preventive care, particularly for the detection of arrhythmias in refining cardiovascular care [3]. Therefore, this review focuses on the management of arrhythmias using AI in modern cardiovascular medicine. It explores the challenges associated with arrhythmia detection, evaluates ECG interpretation algorithms, and discusses predictive models that enhance the clinical decision-making process. Limitations, such as regulatory and workflow considerations, are also addressed in this review. Furthermore, novel methods for proactive and individualized arrhythmia management are emphasized, including wearable technology, real-time monitoring, and cardiac digital twins. Overall, this paper reviews the current evidence to illustrate how integrating AI into arrhythmia care has the potential to close diagnostic gaps, support more informed clinical decision-making, and pave the way for a future of personalized, proactive, and outcome-driven cardiovascular management.

## Review

Methodology

A comprehensive literature search was conducted to identify relevant studies discussing the integration of AI in arrhythmia diagnostics and management. The primary databases searched included PubMed, Scopus, Web of Science, and Google Scholar. The search strategy utilized combinations of the following keywords: “Artificial Intelligence,” “Machine Learning,” “Deep Learning,” “Arrhythmia,” “Cardiovascular Disease,” and “ECG Interpretation.” The search was restricted to articles published between 2015 and 2025 to ensure the review focused on recent technological advancements. Only peer-reviewed journal articles, clinical trials, and comprehensive reviews published in English were included. Studies that did not directly address cardiovascular care, non-English literature, and non-peer-reviewed preprints were excluded from this review.

The role of AI in modern diagnostics

Artificial intelligence has progressed from computer science-based models to clinical tools that help with modern diagnostics, particularly in cardiology. Initial rule-based systems have now developed into ML and DL tools capable of combining large amounts of clinical data to guide treatment plans. Notable recent developments include improvements in algorithms, processing power, and data accessibility, which have played a significant role in assimilating AI into cardiovascular diagnostics [4]. In clinical medicine, AI can assist with precision diagnostics, prognostics, and decision-making by analyzing imaging, ECGs, genomic data, and electronic health record data [5]. Some of the early applications include ECG and MRI interpretation, with diagnostic accuracy comparable to that of experts in detecting arrhythmias, ischemic alterations, and ventricular failure [6]. In addition, DL has improved these functions to identify features, such as those seen in heart failure with preserved ejection fraction, which are harder to localize, helping to form the basis of personalized treatment plans [5]. Recent advancements have shown a shift from population-based prediction to tailored, data-driven cardiovascular treatment. AI models now incorporate multimeric data, cardiac MRI, and echocardiographic strain analysis to improve the diagnostic and prognostic accuracy [4,5]. DL-based imaging enables the detection of minute abnormalities that are undetectable by the eye through the analysis of cardiac anatomy [4]. Additionally, AI-supported clinical decision systems aid in patient triage and the management of acute coronary syndromes; however, evidence demonstrating that improved algorithmic accuracy translates into better patient outcomes is still limited [6]. Therefore, AI in cardiology has evolved from being a theoretical innovation to the application of its technology in daily clinical scenarios, while emphasizing the importance of careful ethical consideration-based implementation [4,6]. Furthermore, AI is increasingly being incorporated into predictive and preventive medicine in cardiology. AI applications in electrophysiology, angiography, imaging, biomarkers, and genomes allow rapid interpretation of multimodal data. ML and DL algorithms can identify intricate disease patterns that conventional models fail to detect, improving the early detection of cardiovascular risk through improved visualization of coronary lesions and plaque structures [7]. In preventive cardiology, AI supports risk prediction and lifestyle-based prevention strategies. AI systems can detect and monitor variables such as diet, physical activity, hypertension, dyslipidemia, sleep, and mental health [8]. Smartphone applications and smartwatches offer customized settings and education to aid in early risk detection. AI uses a combination of physiological and behavioral data to continuously track information to improve overall patient-focused cardiovascular care.

Current challenges in arrhythmia detection and management

Although there have been many remarkable developments and breakthroughs in cardiovascular care, the clinical field still faces some limitations due to issues such as delayed diagnosis, inconsistent interpretation, and, most importantly, technological limitations. These obstacles make it difficult to identify arrhythmias and deliver the best possible treatment. Above all, in certain circumstances, paroxysmal or asymptomatic arrhythmias are extremely challenging to identify at an early stage. Unfortunately, patient outcomes and survival rates may be affected, leading to patient suffering as a result of these restrictions. A recent study found that the short period and length of time of ECG monitoring and variable and unrecognizable symptom presentations can contribute greatly to delayed arrhythmia diagnosis, which can result in a lower survival rate and less effective therapy. Short-term ambulatory ECG monitors can identify only a very small fraction of transient rhythm irregularities, leading to false negatives and delaying the start of treatment. Furthermore, even when arrhythmias are detected, clinicians continue to have difficulty interpreting ECG tracings, leading to different and irregular conclusions [9]. An interesting study found that many cardiologists had high variability in detecting atrial fibrillation (AF) and other complex rhythm abnormalities. These variations may result in inaccurate diagnoses and below-average treatment decisions [10]. The recurrent nature of these challenges highlights the unique and specific flaws in the diagnostic models currently in use. Even if more modern technologies, such as continuous wearable ECG patches and AI-integrated rhythm analysis, show promise, they are still affected and controlled by problems such as data quality, cost, and the need for sufficient specific populations [11]. The complexity of each patient's personal variables, including comorbidities and the type of arrhythmia, will most likely determine the most effective course of treatment, making providing care more challenging than just the simple identification of the arrhythmia. In contrast, adding ML tools to remote monitoring systems may increase the precision of diagnosis by constantly learning and advancing beyond the limitations of human errors and capabilities. However, in the future, this development might raise some ethical concerns regarding the safety of the algorithm and the procedure in use, and the security of everyone's personal data. To address these major issues, an organized approach that requires more physician education, greater access to monitoring tools, and the incorporation of AI into clinical practice is crucial. One might say that, in the future, accurate, targeted arrhythmia detection is quite achievable for all, but it will definitely require constant, dedicated innovation, collaboration, and consideration of upcoming emerging new technologies.

AI algorithms in ECG interpretation

Computerized interpretation of ECGs (CIE) is a ubiquitous tool that has served physicians for many years; however, because it relies on predefined rules and algorithms designed by humans, CIE has its shortcomings [12]. AI can overcome these limitations. AI is a computer model that can mimic human cognition because it uses known information to make decisions and improve its performance with increased experience [13,14]. AI includes ML and DL. ML and DL differ from overarching AI because they rely on data rather than a human-generated set of rules to compute solutions. ML and DL identify patterns between inputs and outputs and can apply these novel algorithms to new situations. ML relies on manually chosen important information to make predictions, whereas DL uses artificial internal neural networks to “train” by identifying patterns without manual selection [13,15]. Training is performed via the identification and extraction of features. DL reduces bias and provides predictions based on ECG data that human physicians cannot [16]. Many different DL models exist, but the most widely used in ECG analysis are convolutional neural networks (CNN), residual networks (ResNet), long short-term memory architecture (LSTM), and transformers. CNN is an architecture in which its convolutional layers are designed for pattern recognition, whereby they extract features from the input data, which is especially useful in ECG interpretation [12,17,18]. ResNet is a type of CNN that is used because it reduces the vanishing gradients and degradation of data, and LSTM addresses the vanishing gradient issue by adding a block called a “Memory Cell” to the model. This block stores data until they are ready to be retrieved [13,18,19]. The transformer network is a model that captures temporal features using an attention-based mechanism. Transformers are useful when used in conjunction with CNNs [18,20]. The process of developing a DL model to evaluate ECG data (AI-ECG) is twofold: the training phase, which includes internal validation, and the testing phase, which is, external validation. Different metrics, such as accuracy, sensitivity, specificity, F1-score, and area under the curve (AUC), were assessed to determine how well this model can perform with real-world data [16]. Numerous AI-ECG models have been published in the scientific literature that can identify, diagnose, or predict patients at risk for various arrhythmias. A summary is included in Table 1, outlining the AI models and basic overviews of the studies.

**Table 1:** Summary of Published AI-ECG Models DL, deep learning; NSR-ECG, normal sinus rhythm ECG; AF, atrial fibrillation; CNN, convolutional neural network; MIT-BIH, Massachusetts Institute of Technology-Beth Israel Hospital Arrhythmia Database; PVC, premature ventricular contraction; DB, discrete heartbeats; ESUS, embolic stroke of undetermined source; AUC, area under the curve; LSTM, long-short-term memory; ICM, insertable cardiac monitor.

Reference	Dataset	Purpose of Study	Model ECG Inputs	DL Model Type	Arrhythmia	Results	Conclusion
[10]	Mayo Clinic Digital Data Vault (180,922 patients with 649,931 ECGs)	Usage of NSR-ECG readings in patients with subsequent AF to detect an AF ECG signature that can be used to predict future AF in asymptomatic patients	12-Lead ECG	CNN	AF	AUC = 0.90; sensitivity = 82.3%; specificity = 83.4%; F1 = 45.4%; accuracy = 83.3%	AI-ECG can be used to screen NSR-ECG in patients with risk for AF and potentially initiate prophylactic anti-coagulation to prevent ESUS
[21]	MIT-BIH (44,103 normal and 6423 PVC beats); INCART (106,239 normal and 9987 PVC beats)	Identification of PVCs by the ResNet-18 using transfer learning model from ECG data	ECG	ResNet-18	PVC	Accuracy: 99.7% (MIT-BIH) and 99.9% (INCART)	The model with great accuracy cross-validated PVCs in the unseen MIT-BIH and INCART data
[22]	326,904 ECGs acquired from two Ewha Womans University Hospitals	Three DL models based on DB and 12-lead ECG data predict arrhythmia occurrence	12-Lead ECG, DB	ResNet, LSTM, Transformer	AF/Multiple arrhythmias	LSTM trained on DB predicted AF and multiple arrhythmias, AUC = 0.92; LSTM trained on 12-lead ECG predicted AF and multiple arrhythmias, AUC = 0.89	DL models predict AF and multiple arrhythmias more effectively utilizing DBs than models using entire 12-lead ECG, suggesting an existence of biomarkers indicative of arrhythmias in NSR-ECGs
[23]	737,815 NSR-ECGs	Usage of NSR-ECG to diagnose asymptomatic AF in patients post-ESUS	12-Lead ECG	Transformer	AF	58/353 (14.4%) post-ESUS patients were identified by ICM for having AF. AF identification by AI-ECG is AUC = 0.81 (no clinical parameters)	AI-ECG has good prognostic value in predicting AF in post-ESUS patients, potentially improving secondary prevention of further strokes

Improving clinical decision-making through predictive modeling 

Predictive modeling for arrhythmia treatment has attracted tremendous interest as a tool for enhancing clinical decision-making through risk stratification and management guidance. ML is preferred over traditional clinical scores because it does not rely on fixed variables or linear approximations of the data. A study demonstrated that ML models substantially outperform traditional scores across different patient groups and methods, such as CNNs and LSTM, which have demonstrated superior prediction for ventricular arrhythmia [24]. This allows for the more precise identification of high-risk patients who may benefit from an implantable cardioverter-defibrillator (ICD) or subsequent follow-up. Predictive modeling also aims to anticipate impending arrhythmic events. ECG time-series data can be incorporated with mechanistic modeling to create a model for predicting malignant arrhythmias in ICD patients [25]. Another study showed similar ML-embedded simulations that demonstrated individual myocyte-level arrhythmia susceptibility more accurately than markers such as the QT interval [26]. These approaches indicate a move away from traditional risk factors toward the dynamic modeling of electrophysiological behavior and interactions. However, it has been pointed out that these models have not been widely used in clinical practice because they are not prospectively validated but rather developed and assessed as retrospective data tools with interpretability problems and a lack of regulatory agreement [24]. It can be argued that models will not exhibit clinical effectiveness until there are larger datasets, accurate interpretations, and strong pathways to incorporate predictive power into medicine [27]. Moreover, predictive modeling has already been used in the care of patients with AF, as confirmed by the electronic health record-atrial fibrillation (EHR-AF) score, which was more accurate than traditional methods in differentiating between patients and examined arterial stiffness and ECGs to find an AUC of 0.789 for early AF symptoms [28,29]. Modeling and data can enable earlier identification and prevention, enhance arrhythmia care, and enable us to better stratify risk, predict when problems might begin, and detect them at earlier stages. This realization requires validation, clear interpretation, and incorporation into clinical workflows.

Integrating AI into clinical workflows: Opportunities and barriers

There are many benefits to integrating AI into electronic health records, such as better communication and support in decision-making. This has been demonstrated by AI models applied to standard 12-lead ECGs to detect low left ventricular ejection fractions (LVEF) [30]. An ECG-AI has been integrated into the EHR system at the Mayo Clinic, displaying both ECG and AI probabilities, and this system is beneficial for the early diagnosis of mildly symptomatic patients [31]. Although there are benefits, obstacles remain. Cost is a topic widely discussed in the context of AI integration. This is highlighted by the PULSE-AI trial, which showed that hospitals benefit financially from improved detection capability but may not account for significant startup costs that can impact a smaller institution [32]. Another topic is unexplainable AI, where the reasoning behind certain decisions is not accessible to the doctor, which can be an obstacle in the clinical workflow [33]. There are many approaches to making AI explainable, each with its own strengths and weaknesses, including methods such as saliency mapping, which allows for viewing the segments most influential in predictions and provides some visibility [28,34]. In addition, predictions generalizable across various ECG lead placements, systems, and more may require greater validation despite early evidence suggesting that predictive power may be retained [28,30]. Issues such as regulations and ethics further complicate AI integration. FDA regulatory behaviors, such as classifying AI tools under its Software as a Medical Device (SaMD), have been inconsistent and not very transparent [35]. Ethically, strict government oversight of patient data and patient transparency regarding the decision to use AI care are important [36].

Future directions: Toward personalized and proactive arrhythmia care

By transitioning from short-duration ECG monitoring to continuous ECG monitoring throughout the clinical setting, as well as population-level rhythm surveillance, AI combined with patient electronic devices is significantly changing the arrhythmia detection capabilities. Therefore, instead of using traditional evaluation, smartwatches, patches, and single-lead ECGs, we would be able to detect paroxysmal and asymptomatic arrhythmias with much higher sensitivity and specificity, allowing for an earlier diagnosis [37,38]. Although this change could possibly enhance testing with continuous evaluation, there are still many significant issues to keep in mind along the way. Some of these issues include incomplete data integration, unknown systems used for device alarms, and an increase in the number of false positives. Research helps prioritize validated algorithms, telehealth workflows, and the implementation of unique and specific trials to assess the best clinical outcomes, such as reductions in stroke incidence and hospitalization [37]. Real-time monitoring represents an additional advantage in the field of AI. Multimodal sensors, such as continuous ECG, photoplethysmography (PPG), heart rate variability, and activity data, can be processed by more optimized AI to generate almost instant alerts in real time [38]. This adds supporting evidence for the rapid initiation of anticoagulation therapy for AF or urgent management of malignant arrhythmias. Some new systems have demonstrated a higher frequency for safer outpatient monitoring and automated triage but still face challenges, including signal quality, specificity, and cybersecurity. Current evidence focuses heavily on algorithm accuracy rather than clinical effectiveness; as such, future trials must determine whether real-time alerts actually improve outcomes, such as arrhythmia-related concerns, emergency visits, and most importantly, mortality [38,39]. Individualized, simulation-based therapy planning is possible with cardiac digital twins, which are personalized 3D virtual models of a patient’s heart built from imaging, electrophysiology, wearable, and genetic data [40,41]. According to some studies, depicting the exact pro-arrhythmic substrates and evaluating medication or ablation techniques can potentially drastically increase accuracy and reduce recurrence in the future. However, high computational requirements, inconsistent data standards, and constantly changing legal frameworks limit the desired level of integration [40]. Some of this desired integration is made possible by the incorporation of digital ECG, wearables, and AI-integrated models. These models may significantly improve decision-making regarding the choice of anticoagulation, specific medication, and ablation technique for each patient [40]. However, rather than focusing only on patient outcomes, the accuracy of these measurements continues to heavily influence the projected evidence and its acceptance by the majority. To achieve the desired outcomes, randomized trials comparing AI-based approaches with guideline-based therapies are required. Finally, resolving biases in some training datasets, workflow integration, addressing reimbursement issues, and addressing privacy concerns are necessary for balanced implementation [39]. To guarantee safe and significant clinical uptake, extensive and versatile implementation studies and standardized validation procedures are crucial for future AI use.

## Conclusions

Artificial intelligence is rapidly transforming the way we treat patients with arrhythmias by improving diagnostic accuracy, risk prediction, and enabling more proactive and individualized management. AI-driven tools in modern cardiology have advanced from experimental computational systems to clinically important technologies that can combine many physiological and behavioral datasets, identify tiny imaging signals, and decipher complex ECG patterns. These capabilities address long-standing gaps in arrhythmia detection, including short monitoring times, delayed paroxysmal rhythm diagnosis, and inconsistent clinician interpretation. Even when arrhythmia is not clinically evident, DL models have shown excellent performance in detecting AF, premature ventricular contractions, and other rhythm abnormalities. By describing dynamic risk instead of relying on static clinical scores, predictive modeling can further improve care. These approaches may help predict malignant arrhythmias, enhance ICD implantation choices, and aid early detection of AF. However, clinical adoption is hampered by problems with external validation, transparency, regulatory guidance, and integration into routine workflows. Strong data governance, transparent and understandable tactics, and alignment with electronic health records are necessary for effective implementation of AI in healthcare. The landscape of arrhythmia is likely to change significantly in the future. While continuous monitoring with smartwatches, patches, and multimodal sensors has made arrhythmia detection a part of everyday life, real-time analytics may enable timely responses to high-risk situations. Cardiac digital twins enable patient-specific simulations that guide customized therapy planning. Despite these advancements, the healthcare industry still needs to ensure fair access to new technologies, address any biases, and prioritize clinical outcomes over algorithmic accuracy. AI offers a powerful path for timely, reliable, and customized arrhythmia treatment. Continued interdisciplinary collaboration, rigorous validation, and ethical implementation will determine the extent to which these tools translate into improved patient outcomes on a broad scale.
